# Analysis of gene mutations associated with isoniazid, rifampicin and ethambutol resistance among *Mycobacterium tuberculosis *isolates from Ethiopia

**DOI:** 10.1186/1471-2334-12-37

**Published:** 2012-02-10

**Authors:** Belay Tessema, Joerg Beer, Frank Emmrich, Ulrich Sack, Arne C Rodloff

**Affiliations:** 1 Department of Medical Laboratory Technology, College of Medicine and Health Sciences, University of Gondar, Gondar, Ethiopia; 2 Institute of Medical Microbiology and Epidemiology of Infectious Diseases, University Hospital of Leipzig, Leipzig, Germany; 3 Institute of Clinical Immunology, University Hospital of Leipzig, Leipzig, Germany; 4 Fraunhofer Institute for Cell Therapy and Immunology, Leipzig, Germany

**Keywords:** *Mycobacterium tuberculosis*, Drug resistance, Gene mutation

## Abstract

**Background:**

The emergence of drug resistance is one of the most important threats to tuberculosis control programs. This study was aimed to analyze the frequency of gene mutations associated with resistance to isoniazid (INH), rifampicin (RMP) and ethambutol (EMB) among *Mycobacterium tuberculosis *isolates from Northwest Ethiopia, and to assess the performance of the GenoType^® ^MTBDRplus and GenoType^® ^MTBDRsl assays as compared to the BacT/ALERT 3D system.

**Methods:**

Two hundred sixty *Mycobacterium tuberculosis *isolates from smear positive tuberculosis patients diagnosed between March 2009 and July 2009 were included in this study. Drug susceptibility tests were performed in the Institute of Medical Microbiology and Epidemiology of Infectious Diseases, University Hospital of Leipzig, Germany.

**Results:**

Of 260 isolates, mutations conferring resistance to INH, RMP, or EMB were detected in 35, 15, and 8 isolates, respectively, while multidrug resistance (MDR) was present in 13 of the isolates. Of 35 INH resistant strains, 33 had mutations in the *katG *gene at Ser315Thr 1 and two strains had mutation in the *inhA *gene at C15T. Among 15 RMP resistant isolates, 11 had *rpoB *gene mutation at Ser531Leu, one at His526Asp, and three strains had mutations only at the wild type probes. Of 8 EMB resistant strains, two had mutations in the *embB *gene at Met306Ile, one at Met306Val, and five strains had mutations only at the wild type probes. The GenoType^® ^MTBDRplus assay had a sensitivity of 92% and specificity of 99% for INH resistance, and 100% sensitivity and specificity to detect RMP resistance and MDR. The GenoType^® ^MTBDRsl assay had a sensitivity of 42% and specificity of 100% for EMB resistance.

**Conclusion:**

The dominance of single gene mutations associated with the resistance to INH and RMP was observed in the codon 315 of the *katG *gene and codon 531 of the *rpoB *gene, respectively. The GenoType^® ^MTBDRplus assay is a sensitive and specific tool for diagnosis of resistance to INH, RMP and MDR. However, the GenoType^® ^MTBDRsl assay shows limitations in detecting resistance to EMB.

## Background

According to the World Health Organization (WHO) report, the proportion of multidrug resistant tuberculosis (MDR-TB), resistant to at least isoniazid and rifampicin among new and previously treated TB cases globally ranges from 0% to 28.3% and from 0% to 61.6%, respectively [1]. In Ethiopia, the countrywide anti-TB drug resistance survey conducted in 2005 showed that the prevalence of MDR-TB was 1.6% and 11.8% among new and previously treated TB cases, respectively [2]. Moreover, 5825 MDR-TB cases were estimated to have occurred in 2006 in Ethiopia [3]. MDR-TB treatment involves prolonged use of second-line anti-TB drugs that are less effective, less tolerated, more toxic, and more expensive than first-line anti-TB drugs [4]. In most high-burden TB countries, MDR-TB is only diagnosed after prolonged treatment with first-line TB drugs and clinical recognition that treatment has failed. Treatment of drug-resistant TB with standard first-line drugs, instead of a regimen designed according to the resistance pattern has several potential adverse consequences: patients remain on inadequate treatment longer, increasing the risk of treatment failure or death; selection of drug resistant strains and patients remain infectious, promoting transmission to close contacts [5].

In Ethiopia, the treatment regimens for category I and category II (retreatment regimen) tuberculosis cases are 2 (RMP-INH-EMB-PZA)/4(RMP-INH) and 2 STM (RMP-INH-EMB-PZA)/1(RMP-INH-EMB-PZA)/5(EMB_3 _(RMP-INH)_3_), respectively [6]. The standard treatment regimen for MDR-TB is 6(EMB-PZA-KM (AMK)-LFX-ETO-CS)/12(EMB-PZA-LFX-ETO-CS). For proper treatment and control of tuberculosis, WHO is recommending countries to expand their capacity for culture based drug-susceptibility testing (DST) and consider new, molecular-based assays for diagnosing drug resistance [7,8]. Since *M. tuberculosis *usually grows slowly, the identification and drug-resistance testing usually require several weeks. The gold-standard of TB diagnosis by culture takes weeks to become positive, and even with the up-to date automated fluid culture methods it takes an average of 14 days. Another 14 days for additional testing are required to get the information on drug susceptibility [9-11]. Molecular methods of drug resistance testing, based on the identification of mutations in genes associated with drug resistance, like GeneXpert MTB/RIF assay, offer an effective tool for determining drug resistance because of their high sensitivity, specificity and speed [12].

Molecular methods that have been developed to detect drug resistance include the GenoType^® ^MTBDRplus for detection of INH and RMP resistance and the GenoType^® ^MTBDRsl for detection of resistance against EMB, floroquinolones, and aminoglycosides/cyclic peptides (Hain Lifescience, Nehren, Germany). These assays are DNA strip assays that use PCR and hybridization. Mutations in *katG *gene and *inhA *gene were related to the high-level and low-level INH resistance, respectively [13]. Nearly all RMP resistant strains contain mutation of the *rpoB *gene, coding RNA polymerase subunit ß and mutation in the *embB *gene was associated with EMB resistance [14,15].

In Ethiopia, culture and drug susceptibility testing (DST) for *M. tuberculosis *are not performed routinely in clinical microbiology laboratories. Laboratory diagnosis of TB remains in a stage of acid-fast staining. Currently, five regional laboratories are being rebuilt and equipped to perform culture and drug susceptibility testing using methods including GenoType^® ^MTBDRplus assay. The GenoType^® ^MTBDRplus *and *GenoType^® ^MTBDRsl assays have been studied in several laboratories of other countries, however, there is a wide variation in circulating *M. tuberculosis *strains worldwide [16,17], and false negative results may occur due to unique genetic mutations [18-24], affecting the performance of molecular assays for drug susceptibility testing. Therefore, analysis of gene mutations associated with resistance to anti-tuberculosis drugs and assessment of the performance of molecular methods for drug resistance testing in different settings are needed to ensure acceptable performance of the assays. So far, there was no report on the frequency of gene mutations associated with resistance to INH, RMP and EMB and the applicability of these molecular assays for *M. tuberculosis *isolates from Ethiopia. In this study, we analyzed the frequency of gene mutations associated with resistance to INH, RMP and EMB among *M. tuberculosis *isolates from Northwest Ethiopia, and assessed the performance of the GenoType^® ^MTBDRplus for detection of resistance to INH, RMP and MDR and GenoType^® ^MTBDRsl assay for detection of EMB resistance compared to the automated, culture-based, BacT/ALERT 3D system drug susceptibility testing.

## Methods

### Study design, area and study period

Two hundred sixty *M. tuberculosis *isolates from smear positive tuberculosis patients diagnosed between March 2009 and July 2009 at Gondar Hospital, Gondar Health Center, Metemma Hospital, Bahir Dar Hospital and Debre Markos Hospital in Northwest Ethiopia were included in this study. Diagnosis of smear-positive tuberculosis was based on the national guideline for microscopic examination of tuberculosis (6): direct smears were prepared from three sputum specimens and stained by Ziehl-Neelsen staining technique for microscopic examination. Drug susceptibility tests using GenoType^® ^MTBDRplus, GenoType^® ^MTBDRsl and BacT/ALERT 3D system were performed at the mycobacteriology laboratory in the Institute of Medical Microbiology and Epidemiology of Infectious Diseases, University Hospital of Leipzig, Germany. Informed consent was obtained from the study subjects. Institutional ethical clearance was obtained from the research and publication committee of Gondar University, Ethiopia. Details of sputum storage, transportation, isolation and identification of the isolates have described previously [25].

### GenoType^® ^MTBDRplus and GenoType^® ^MTBDRsl drug susceptibility testing

GenoType^® ^MTBDRplus assay for detection of INH and RMP resistance, and GenoType^® ^MTBDRsl assay for detection of ethambutol resistance were performed according to the manufacturer's instructions (Hain Lifescience GmbH, Nehren, Germany). Briefly, DNA was extracted from cultures by heating the bacteria in a heating block for 20 minutes at 95°C followed by sonication in ultrasonic water bath for 15 minutes. Amplification was performed using 2.5 μl (1 unit) *Taq *DNA polymerase (ROCHE, Mannheim, Germany). For the amplification profile the instructions of the manufacturer were followed. Hybridization of the single-stranded, biotin-labeled amplicons to membrane-bound probes on the strip followed by addition of conjugate, and substrate to detect visible band patterns on the strip was performed manually using a shaking water bath, Memmert-SV1422 (Memmert GmbH & CO.KG, Schwabach, Germany) at 45°C. Then strips were allowed to dry and interpreted according to the manufacturer's recommendation.

To detect high level INH resistance, the GenoType^® ^MTBDRplus has incorporated one wild type (WT-315) and two mutation-type probes specific for mutation Ser315Thr1 and Ser315Thr2 of the *katG *gene. For detection of low-level INH resistance this assay has two wild-type probes (WT-15/-16 and WT-8) and four mutation-type probes, covering mutations of C15T, A16G, T8C and T8A in the *inhA *gene. To detect rifampicin resistance, the Genotype MTBDRplus has incorporated eight wild-type probes for the *rpoB *gene, covering codons in the *rpoB *gene from 505 to 533, and four other probes specific for mutations Asp516Val, His526Tyr, His526Asp and Ser531Leu. For detection of ethambutol resistance, the GenoType^® ^MTBDRsl employs one wild-type probe (WT-306) and two mutation probes specific for mutations Met306Ile and Met306Val in the *embB *gene.

### BacT/ALERT 3D system drug susceptibility testing

Drug susceptibility testing for isoniazid, rifampicin and ethambutol was performed by BacT/ALERT 3D system (BioMerieux, S.A, France) according to the methods published previously [26,27]. The final drug concentration in the test bottles was 1 μg/ml for INH and RMP, and 2 μg/ml for EMB. Two control bottles, one with 1% control (0.5 ml of the 1:100 diluted test organisms suspension) and one original control bottle without drug were used for interpretation of the test results. *M. tuberculosis *isolate was determined to be resistant to an antibiotic when the drug-containing bottle had a time to detection (TTD) that was less than or equal to the TTD of the 1% control.

### Statistical analysis

All laboratory data were entered, cleared and analyzed using SPSS version 13 statistical package software (SPSS Inc., Chicago, IL). The standard chi-square tests (*χ*2) were used to assess statistical relationships between predisposing factors and drug-resistant TB. Sensitivity, specificity, positive predictive value and negative predictive value of the molecular methods were analyzed using crosstabulation after arranging the results of the molecular methods in the rows and gold standard, BacT/ALERT 3D system in columns. P values of less than 0.05 were considered statistically significant.

## Results

Of the 260 patients included in this study, the majority of patients, 59% were males. The median age of the study subjects was 28.0 years (range, 7-75 years). History of previous treatment for tuberculosis was significantly associated with gene mutations conferring resistance to INH (*P *= 0.001), RMP (*P *= 0.002) and MDR (*P *= 0.044). HIV co-infection, gender and age of the study subjects had no significant association with gene mutations conferring resistance to INH, RMP and EMB. A summary of patient demographic characteristics and associated drug susceptibility pattern according to the molecular methods is shown in Table 1.

**Table 1:** Characteristics of study subjects and their association with resistance to isoniazid, rifampicin and ethambutol based on GenoType^® ^MTBDRplus and GenoType^® ^MTBDRsl assays

Characteristics	Number of patients	Anti-TB drug resistance							
	
		INH N (%)	P-value	RMP N (%)	P-value	MDR N (%)	P-value	EMB N (%)	P-value
**Gender**									
Male	153	19 (12.4)	0.556	9 (5.9)	0.925	7 (4.6)	0.707	5 (3.3)	0.831
Female	107	16 (15.0)		6 (5.6)		6(5.6)		3 (2.8)	
**Age (years)**									
< 40	214	29 (13.6)	0.927	11 (5.1)	0.348	9 (4.2)	0.205	7 (3.3)	0.696
≥ 40	46	6 (13.0)		4 (8.7)		4 (8.7)		1 (2.2)	
**TB history**									
New	214	22 (10.3)	0.001	8 (3.7)	0.002	8(3.7)	0.044	6 (2.8)	0.582
Previously treated	46	13 (28.3)		7 (15.2)		5 (10.9)		2 (4.3)	
**HIV status**									
Negative	194	29 (14.9)	0.228	9 (4.6)	0.180	9 (4.6)	0.647	7 (3.6)	0.395
Positive	66	6 (9.1)		7 (10.6)		4 (6.1)		1 (1.5)	
**Total**	260	35 (13.5)		15 (5.8)		13 (5.0)		8 (3.1)	

*N *number, *INH *isoniazid, *RMP *rifampicin, *MDR *multidrug resistance, *EMB *ethambutol

### Mutations associated with INH, RMP and EMB resistance

Mutations conferring resistance to isoniazid, rifampicin and ethambutol were detected in 14%, 6% and 3% of the isolates, respectively. Five percent of the isolates showed mutation in both *rpoB *gene and *katG *gene or *inhA *promoter region indicating that they were multidrug resistant. There was no isolate that showed mutations at both *katG *and *inhA *genes. Mutations associated with isoniazid resistance were more often encountered as compared to those seen in rifampicin and ethambutol. Of 35 INH resistant strains, 94% had mutation in the *katG *(codon 315) gene with amino acid change of Ser315Thr1, indicating high level resistance, while 6% of the strains had mutation in the *inhA *gene, C15T, indicating low level resistance. All *katG *gene mutations detected at wild type probes were also present at mutant probes, as was the case with the *inhA *gene mutations (Table 2). Additionally, three strains showing resistance to isoniazid and two strains sensitive to isoniazid by the BacT/ALERT 3D system did not display concordant results by GenoType^® ^MTBDRplus even on repeat assays (Table 3).

**Table 2:** Frequency of gene mutations associated with resistance to isoniazid and rifampicin by GenoType^® ^MTBDRplus, and to ethambutol by GenoType^® ^MTBDRsl assays

Anti TB-drugs	Number of resistant isolates	Patterns of gene mutations (wild-type/mutant)	Amino acid Changes	Frequency (n)
**Isoniazid**	35	*katG*WT/*katG*MUT1	Ser315Thr1	33
		*inhA *WT1/*inhA *MUT1	C15T	2

**Rifampicin**	15	*rpoB *WT8/*rpoB *MUT3	Ser531Leu	11
		*rpoB *WT7/*rpoB *MUT2B	His526Asp	1
		*rpoB *WT2 &3/NA	Unknown	1
		*rpoB *WT4/ND	Unknown	1
		*rpoB *WT6/NA	Unknown	1

**Ethambutol**	8	*embB *WT/*embB *MUT1A	Met306Ile	2
		*embB *WT/*embB *MUT1B	Met306Val	1
		*embB *WT/ND	Unknown	5

*n *number of isolates, *WT *wild-type, *MUT *mutant, *ND *no mutation detected at mutant probe, *NA *mutant probe is not available

**Table 3:** Performance of GenoType^® ^MTBDRplus assay for detection of resistance to INH, RMP and MDR and GenoType^® ^MTBDRsl assay for detection of EMB resistance in comparison to BacT/ALERT 3D system

Molecular methods DST results		BacT/ALERT 3D DST results		Sensitivity %	Specificity %	PPV %	NPV %
						
		Susceptible	Resistant				
INH	Susceptible	222	3	91.7	99.1	94.3	98.7
	Resistant	2	33				

RMP	Susceptible	245	0	100	100	100	100
	Resistant	0	15				

INH +RMP (MDR)	Susceptible	247	0	100	100	100	100
	Resistant	0	13				

EMB	Susceptible	241	11	42	100	100	95.6
	Resistant	0	8				

*DST *drug susceptibility testing, *INH *isoniazid, *RMP *rifampicin, *MDR *multidrug resistance, *EMB *ethambutol, *PPV *positive predictive value, *NPV *negative predictive value

The rifampicin resistant isolates displayed different mutations: 73% of the isolates had mutation at position Ser531Leu, one isolate had mutation at His526Asp, while in three of the isolates mutation was detected only at the wild type probes. Of the isolates with mutation that detected only at wild type probes, one isolate had mutation at *rpoB *WT2 and WT3, one isolate at *rpoB *WT4 and one isolate at *rpoB *WT6. According to the kit manufacturer's recommendation, the three isolates with mutation that detected only at wild type probes were considered resistant (Table 2).

Mutations associated with ethambutol resistance were less frequent compared to those seen in isoniazid and rifampicin resistance. Of the 8 EMB resistant strains according to the molecular method, two strains had mutations in the *embB *(codon 306) gene with amino acid change of Met306Ile and one strain had mutation in the *embB *gene with amino acid change of Met306Val, whereas five strains had mutation that detected only at the wild type probes (*embB *WT) but not at the mutant probes (Table 2). Moreover, 58% of the isolates showing resistance to ethambutol by the BacT/ALERT 3D system did not display a concordant result by GenoType^® ^MTBDRsl assay even on repeat assays (Table 3). All isolates included in this study had no mutations conferring resistance to *fluoroquinolones and aminoglycosides. This might *be due to low use/access to these drugs in Northwest Ethiopia.

### Performance of GenoType^® ^MTBDRplus and GenoType^® ^MTBDRsl assays

Compared with the automated, culture-based, BacT/ALERT 3D system drug susceptibility testing, the GenoType^® ^MTBDRplus assay had a sensitivity of 92% and specificity of 99% for detection of INH resistance, a sensitivity of 100% and specificity of 100% for RMP resistance, and a sensitivity of 100% and specificity of 100% for multidrug resistance. The GenoType^® ^MTBDRsl assay had a sensitivity of 42% and specificity of 100% for detection of EMB resistance (Table 3).

## Discussion

Almost all TB laboratories in Ethiopia have only been equipped with the acid-fast staining and lack resources for culture, identification and drug susceptibility testing of mycobacteria, which present a huge hindrance for tuberculosis control in the country. Therefore, there is an urgent need for laboratories to find a rapid and efficient method for TB diagnosis as a complement to the smear microscopy, and meanwhile to establish MDR-TB diagnostic route for rapid detection of drug-resistant TB. The GenoType^® ^MTBDRplus and GenoType^® ^MTBDRsl assays are rapid and technically simple to perform and do not require sophisticated equipment when compared with the conventional culture-based techniques. These assays have been studied in other countries. However, false negative results reported due to unique genetic mutations associated with resistance to anti-tuberculosis drugs in different countries [18-24]. Therefore, in this study, we investigated the frequency of gene mutations associated with resistance to INH, RMP and EMB and evaluated the performance of these molecular assays for detection of resistance to INH, RMP and EMB on *M. tuberculosis *isolates from Northwest Ethiopia.

In this study, the GenoType^® ^MTBDRplus assay had a sensitivity of 92% and specificity of 99% for INH resistance, and 100% sensitivity and specificity for RMP resistance and for multidrug resistance. Other reports have shown that the performance of the GenoType^® ^MTBDRplus assay in sensitivity and specificity almost comes up to that of conventional culture-based susceptibility testing: Causse et al. [28] reported a sensitivity of 95% for INH and 100% for RMP, a Meta analysis report by Bawanga et al. [29] showed that GenoType^® ^MTBDRplus assay has a sensitivity and specificity of 96% and 100% for INH and 99% and 99% for RMP, respectively. In the present study, 8% of phenotypically defined isoniazid-resistant strains had no mutations in codon 315 of the *katG *gene and in the regulatory region of the *inhA *gene, demonstrating that other mechanisms or mutations in other codons of the katG gene may be responsible for the development of INH resistance in *M. tuberculosis *strains. Interestingly, all phenotypically defined rifampicin-resistant strains and multidrug-resistant strains had mutations conferring resistance to rifampicin, and both isoniazid and rifampicin resistance (MDR). Suggesting that the set of the DNA probes used in the GenoType^® ^MTBDRplus assay covers most of the mutations prevalent in Northwest Ethiopia. However, previously reported associations between the gene mutations and Beijing strains [30,31] suggest that the assay may be potentially useful in the area with a high prevalence of Beijing family (Eastern Europe, China and South-East Asia).

Previous studies have shown that 40-95% of isoniazid resistance are defined as the high-level drug-resistance due to the *katG *gene mutations. Of which, 75-90% are recognized as mutations in the 315th codon of the *katG *gene, which mainly result in Ser315Thr1 and Ser315Thr2 mutation [13,15,32]. In the present study, 94% of INH resistances, close to the high limit of reported range, were attributed to *katG *mutations which confer high level resistance to INH. Of which, 100% were identified as Ser315Thr1 mutation. Studies have also shown that 8% to 43% of INH resistance are defined as the low-level drug resistance mainly caused by the mutations in the promoter region of *inhA *gene [33]. In this study, we have observed that the low-level drug-resistance proportion was 6%, close to the low limit of the reported range.

In the previous studies [14,15,34], about 95% of resistance to RMP are associated with the *rpoB *gene mutations which are found to cluster mainly in the region of codon 507-533. In this study, the distribution of gene mutation among RMP resistant isolates was 73% at position Ser531Leu and 7% at His526Asp. In 20% of the resistant isolates, mutation was detected only at the wild type probes, which is different from the previously reported gene mutation distribution in China, 37% at Ser531Leu, 3% at His526Asp and in 60% of the isolates, mutation was detected only at the wild type probes [35], reflecting the difference in the distribution of gene mutations associated with RMP resistance in different geographical locations. The high frequency (20%) of RMP resistant isolates with no mutation at the mutant probes, probably indicating the presence of less common mutations at *rpoB *gene that can not be detected by the current version of the GenoType^® ^MTBDRplus assay.

In this study, the distribution of gene mutation among 8 isolates showing resistance to EMB by GenoType^® ^MTBDRsl assay was 25% at Met306Ile, 13% at Met306Val and 63% of the strains had mutation only at the wild type probes. Furthermore, 58% of the isolates showing resistance to ethambutol by the BacT/ALERT 3D system did not display a similar result by this molecular assay even on repeat assays. Consequently, in the present study, GenoType^® ^MTBDRsl assay had sensitivity of 42% and specificity of 100% for ethambutol resistance. Similarly, other previous studies have shown that this assay has low sensitivity, only about 50% for detection of EMB resistance [36-38]. The present study together with previous reports, highlight the fact that the molecular basis of EMB resistance in *M. tuberculosis *is still insufficiently understood to allow detection of EMB resistance by molecular methods.

## Conclusions

In our study, the dominance of single gene mutations associated with the resistance to isoniazid and rifampicin in the codon 315 of the *katG *gene and codon 531 of the *rpoB *gene was observed. The GenoType^® ^MTBDRplus assay is a sensitive and specific tool for diagnosis of resistance to INH, RMP and MDR. The short turnaround times and the potential for rapid screening of large numbers of isolates make it suitable as a first-line screening assay for TB drug resistance. Its application and popularization will help better solve the long-standing problem of laboratory diagnosis of drug resistance in Ethiopia. In the majority of phenotypically ethambutol resistant isolates, gene mutation associated with the resistance to ethambutol was not detected by this assay. This indicates that the present version of the GenoType^® ^MTBDRsl assay shows limitations in detecting resistance to ethambutol. Further studies are required to understand the mechanism of resistance to ethambutol and to evaluate GenoType^® ^MTBDRplus assay for the diagnosis of INH and RMP resistance from direct sputum specimens of tuberculosis patients in Ethiopia.

## Competing interests

The authors declare that they have no competing interests.

## Authors' contributions

BT was the primary researcher, conceived the study, designed, participated in sample collection, performed laboratory experiments, conducted data analysis and drafted the manuscript for publication. JB participated in doing the laboratory experiments, interpreting the results, and reviewed the initial and final drafts of the manuscript. FE, US and AR reviewed the initial and final drafts of the manuscript. All authors read and approved the final manuscript.

## Pre-publication history

The pre-publication history for this paper can be accessed here:

http://www.biomedcentral.com/1471-2334/12/37/prepub
